# Voltammetric Response of Alizarin Red S-Confined Film-Coated Electrodes to Diol and Polyol Compounds: Use of Phenylboronic Acid-Modified Poly(ethyleneimine) as Film Component

**DOI:** 10.3390/s18010317

**Published:** 2018-01-22

**Authors:** Shigehiro Takahashi, Iwao Suzuki, Takuto Ojima, Daichi Minaki, Jun-ichi Anzai

**Affiliations:** 1 Faculty of Pharmacy, Takasaki University of Health and Welfare, 37-1 Nakaorui, Takasaki 370-0033, Japan; takahashi-shi@takasaki-u.ac.jp (S.T.); suzuki@takasaki-u.ac.jp (I.S.); 2 Graduate School of Pharmaceutical Sciences, Tohoku University, Aramaki, Aoba-ku, Sendai 980-8578, Japan; b4yB1012@s.tohoku.ac.jp (T.O.); daichi.minaki.e3@tohoku.ac.jp (D.M.)

**Keywords:** voltammetric response, layer-by-layer film, alizarin red S, phenylboronic acid

## Abstract

Alizarin red S (ARS) was confined in layer-by-layer (LbL) films composed of phenylboronic acid-modified poly(ethyleneimine) (PBA-PEI) and carboxymethylcellulose (CMC) to study the voltammetric response to diol and polyol compounds. The LbL film-coated gold (Au) electrode and quartz slide were immersed in an ARS solution to uptake ARS into the film. UV-visible absorption spectra of ARS-confined LbL film suggested that ARS formed boronate ester (ARS-PBS) in the film. The cyclic voltammetry of the ARS-confined LbL film-coated electrodes exhibited oxidation peaks at −0.50 and −0.62 V, which were ascribed to the oxidation reactions of ARS-PBS and free ARS, respectively, in the LbL film. The peak current at −0.62 V increased upon the addition of diol or polyol compounds such as L-dopa, glucose, and sorbitol into the solution, depending on the concentration, whereas the peak current at −0.50 V decreased. The results suggest a possible use of ARS-confined PBA-PEI/CMC LbL film-coated Au electrodes for the construction of voltammetric sensors for diol and polyol compounds.

## 1. Introduction

**Figure 1:**
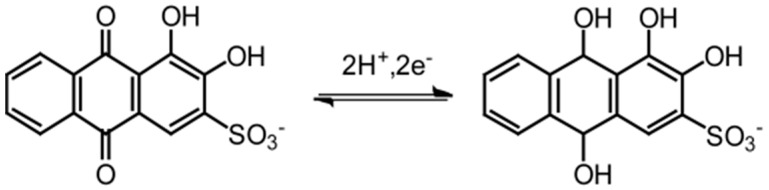
Redox reactions of ARS.

The Polyelectrolyte multilayer films prepared by a layer-by-layer (LbL) deposition technique have attracted much attention because of their facile preparation and potential applications to thin film devices [1,2,3,4,5]. A variety of polyelectrolytes can be used as materials for the preparation of LbL-deposited films, which include synthetic polymers [6,7], biopolymers [8,9], and carbon and metal nanomaterials [10,11]. Consequently, LbL films are currently widely used for the development of surface coatings [12], drug delivery systems [13], biosensors [14] and so forth. In this context, we have recently prepared redox-active LbL films that contain alizarin red S (ARS) (Figure 1) [15]. ARS was confined in the LbL films composed of poly(ethyleneimine) (PEI) and carboxymethylcellulose (CMC) through an electrostatic affinity between negatively charged ARS and positive charges in the PEI/CMC films. The ARS-modified PEI/CMC film-coated gold (Au) electrodes exhibited electrochemical responses originating from the redox reactions of the 9,10-anthraquinone moiety in ARS (Figure 1). The electrodes showed two oxidation peaks in the cyclic voltammogram (CV) recorded in phenylboronic acid (PBA) solutions, which originated from the boronate ester of ARS (ARS-PBA) and free ARS in the film. The results demonstrated that ARS binds PBA to form ARS-PBA in the LbL film [16]. Furthermore, the intensity of the redox peak originating from the ARS-PBA decreased when diol compound was added to the solution [15]. The results suggested a potential use of the ARS-confined PEI/CMC film-coated electrodes for the voltammetric detection of diol compounds. 

**Figure 2:**
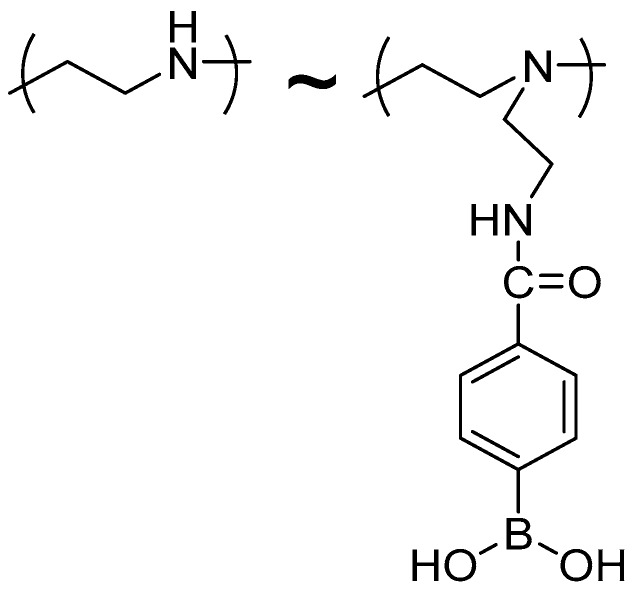
Chemical structure of PBA-PEI.

A drawback of the PEI/CMC film-coated electrodes was that PBA must be added to the sample solutions for the voltammetric measurements. The voltammetric response of the PEI/CMC film-coated electrodes depended on the concentration of added PBA. In addition, the response mechanism of the electrodes was rather complicated, because the binding equilibria between PBA and diol compound both in the film and in the solution concurrently affected the redox response. Consequently, reagentless sensors that can be operated without adding PBA are desirable from a practical viewpoint. Therefore, in this study, we used PBA-modified PEI (PBA-PEI) (Figure 2), in which PBA moieties are covalently linked to the PEI chain through amide bonds, as a component of LbL film to construct PBA-PEI/CMC film-coated Au electrodes, enabling the measurements to be carried out without adding PBA in the sample solutions.

## 2. Materials and Methods

### 2.1. Materials

An aqueous solution of PEI (30%, molecular weight (MW): 60,000–80,000) was purchased from Nacalai Tesque Co. (Kyoto, Japan). PEI has a random branched structure with the ratio of primary, secondary, and tertiary amino groups being nominally about 1:2:1. CMC was obtained from Tokyo Kasei Co. (Tokyo, Japan). Sodium 3-mercapto-1-propanesulfonate (MPS) was obtained from Sigma-Aldrich Chemical Co. (St. Louis, MO, USA). ARS was from Nacalai Tesque Co. All chemicals were of reagent grade and used as received. 4-Carboxyphenylboronic acid *n*-propylamide (4CPBA-PA) was purchased from Combi-Block Inc. (San Diego, CA, USA). A commercially-available Au disc electrode (diameter, 3 mm, BAS Co. Ltd., Tokyo, Japan) was used throughout.

PBA-PEI was synthesized by reacting 4-carboxyphenylboronic acid and PEI in the presence of 1-ethyl-3-(3-dimethylaminopropyl)carbodiimide hydrochloride in water and purified by dialysis according to the reported procedures [17]. Thus, PBA-PEI bearing 24 mole% PBA was obtained, in which PBA residues were attached to 24 mole% of the total amino groups of PEI. The PBA content was determined based on the intensity of the absorption band at 243 nm originating from the PBA residue. In order to calculate the PBA content in PBA-PEI, we used the molar extinction coefficient of a model compound (4CPBA-PA) (*ε*, 13,000 at 243 nm), assuming that the ε value of PBA residue in PBA-PEI is comparable to that of the model compound.

### 2.2. Methods

LbL films composed of PBA-PEI and CMC were coated on the surface of the Au disc electrode which had been polished with alumina slurry and thoroughly rinsed in deionized water. The surface of the electrode was cleaned by scanning an electrode potential from −0.2 to +1.5 V at a scan rate of 0.1 V∙s^−1^ in 0.5 M H_2_SO_4_. The clean electrode was first modified with MPS by treating the electrode in aqueous 10 mM MPS solution overnight. Then, the MPS-modified Au electrode was alternately immersed in 0.1 mg∙mL^−1^ PBA-PEI and 0.1 mg∙mL^−1^ CMC solutions (pH 9.0) containing 10 mM NaCl and 10 mM 4-(2-hydroxymethyl)-1-piperazinesulfonic acid (HEPES) for 15 min to deposit the PBA-PEI and CMC. The Au electrode was rinsed in distilled water for 5 min after each deposition. The deposition was repeated to prepare the 10.5-bilayer film. The LbL film-coated electrode was then immersed in a 0.1 mM ARS solution containing 10 mM NaCl and HEPES for 1 h to uptake ARS into the film, followed by immersing the electrode in ARS-free NaCl/HEPES solution overnight to remove weakly bound ARS. 

In order to record UV-visible spectra of the LbL film, the film was deposited on the surface of a quartz slide (50 × 9 × 1 mm^3^), which had been cleaned with a sulfuric acid/chromic acid mixture. The adsorption behavior of ARS into the film was evaluated by recording the UV-visible spectrum of the LbL film-coated slide after immersing the film in the ARS solution. The UV-visible spectra of the films were recorded in 10 mM HEPES solution containing 10 mM NaCl (pH 9.0). The absorption spectra of PBA-PEI and ARS were recorded also in the above solution in the quartz cuvette with a 10-mm light path.

A conventional three-electrode system with a platinum auxiliary electrode and a Ag/AgCl reference electrode was used to record the CVs and DPVs. Thus, the electrode potential reported in this paper always refers to the Ag/AgCl electrode. Sample solutions were purged with nitrogen (N_2_) gas before the electrochemical measurements. All measurements were carried out in the solution containing 10 mM NaCl and 100 mM HEPES at room temperature (ca. 23 °C) under a N_2_ atmosphere. The solutions of pH 9.0 were employed for the electrochemical measurements, because the binding of diol and polyol compounds to PBA is stronger in weakly basic solutions. 

## 3. Results

**Figure 3:**
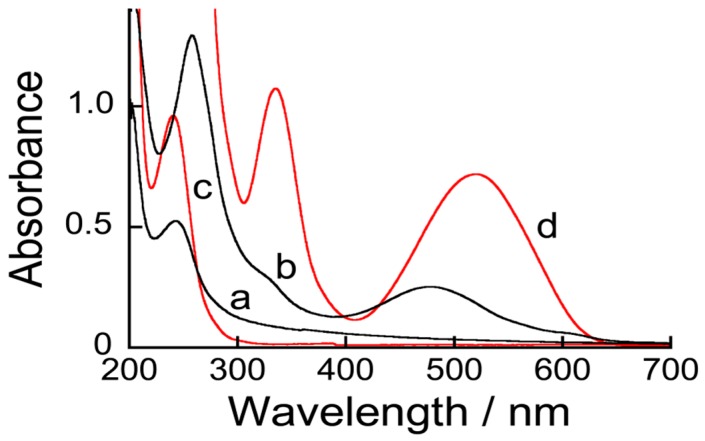
UV-visible absorption spectra of (PBA-PEI/CMC)_10_PBA-PEI film (a); ARS-immobilized (PBA-PEI/CMC)_10_PBA-PEI film (b); PBA-PEI (0.05 mg∙mL^−1^) (c) and ARS (0.1 mM) (d).

**Figure 4:**
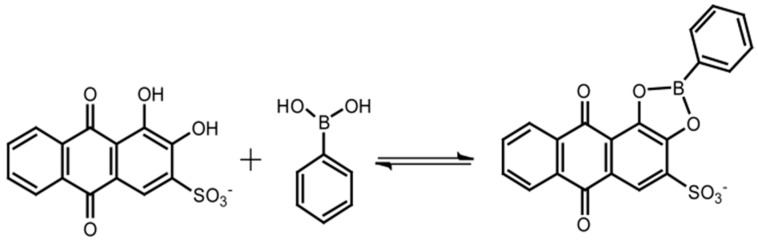
Formation of boronate ester ARS-PBA in the film.

Prior to electrochemical measurements, the 10.5-bilayer (PBA-PEI/CMC)_10_PBA-PEI films were deposited on the surface of a quartz slide to record the UV-visible spectra with and without ARS (Figure 3). The UV-visible spectrum of the film without ARS (spectrum a) exhibited absorption bands at 243 nm originating from PBA-PEI, showing that the LbL films had been successfully prepared on the quartz slide. The electrostatic interactions between PBA-PEI and CMC should be the driving force for the formation of the LbL films. The content of the PBA residue in the 10.5-bilayer (PBA-PEI/CMC)_10_PBA-PEI film was estimated to be 4.0 × 10^−8^ mol∙cm^−2^ from the UV-visible absorption spectrum, using the ε value of 4CPBA-PA. On the other hand, a new absorption band appeared at 477 nm in the UV-visible spectrum of the ARS-immobilized film (spectrum b). It has been reported that ARS shows an absorption band at 510–520 nm in aqueous solutions from pH 6.0 to pH 9.0, while the absorption band exhibits a blue shift when ARS forms boronate ester ARS-PBA [18,19,20]. In fact, we have recorded a UV-visible absorption spectrum of ARS in solution, which exhibited an absorption band at approximately 520 nm (spectrum d). Therefore, the UV-visible spectrum suggests that ARS bound to the PBA residues to form ARS-PBA in the (PBA-PEI/CMC)_10_PBA-PEI film, as illustrated in Figure 4. However, note that the spectroscopic results do not exclude the possibility that a small proportion of free ARS exists in the film, judging by the weak shoulder observed at 500–600 nm. 

**Figure 5:**
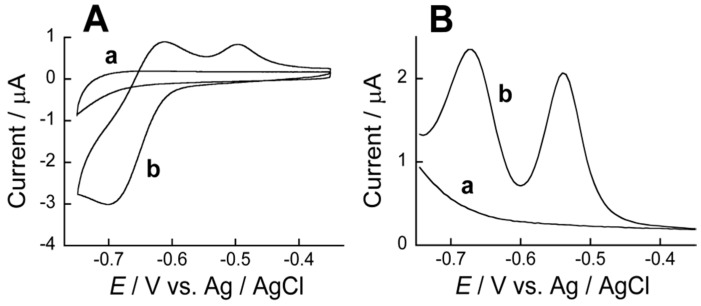
A typical CVs (**A**) and DPVs (**B**) of Au electrodes coated with (PBA-PEI/CMC)_10_PBA-PEI film with (b) and without (a) ARS. CVs were recorded at pH 9.0.

Figure 5 shows CV and differential pulse voltammogram (DPV) of Au electrodes coated with (PBA-PEI/CMC)_10_PBA-PEI film before and after ARS immobilization. No redox peak appeared in the potential range from −0.35 to −0.75 V without ARS. In contrast, two distinct oxidation peaks were observed both in the CV (at −0.50 and −0.62 V) and DPV (at −0.54 and −0.67 V) after immobilization of ARS. The oxidation peaks found at −0.50 V in CV and −0.54 V in DPV may be ascribed to the oxidation reaction of ARS-PBA formed in the film, while the peaks at −0.62 V in CV and −0.67 V in DPV originated from free ARS. These oxidation peaks are resulting from re-oxidation reactions of the reduced forms of ARS-PBA and free ARS. This is because the CVs were recorded by scanning the electrode potential from −0.35 to −0.75 V followed by the reverse scan, while, for the DPVs, the electrode potential was scanned from −0.75 to −0.35 V after the potential was maintained at −0.75 V for several seconds. Similar voltammetric results have been observed for redox reactions of ARS in aqueous solutions [21] and in modified electrodes [22,23,24]. Thus, the CV and DPV results suggest that ARS is immobilized in the (PBA-PEI/CMC)_10_PBA-PEI film in the forms of ARS-PBA and free ARS. It should be noted here that the spectroscopic results suggested most ARS is in the form of boronate ester ARS-PBA in the film. Thus, the results suggest that a limited portion of free ARS and ARS-PBA located adjacent to the electrode surface are involved in the redox reactions in the CV and DPV. In a separate experiment, we evaluated the effect of film thickness on the electrochemical properties using thinner (PBA-PEI/CMC)_5_PBA-PEI film-coated electrode. The redox response of the (PBA-PEI/CMC)_5_PBA-PEI film-coated electrode was found to be rather low as compared to the electrode coated with the (PBA-PEI/CMC)_10_PBA-PEI film, owing to the limited loading of ARS in the thinner film. Therefore, we used the (PBA-PEI/CMC)_10_PBA-PEI film-coated electrode in the following experiments.

It is known that 1,2-dihydroxy groups of ARS can also be electrochemically oxidized [21]. In practice, CV exhibited an oxidation peak at approximately 0.4 V for the ARS-confined (PBA-PEI/CMC)_10_PBA-PEI film-coated electrode, which suggests that the redox response of the ARS-confined electrode shown in Figure 5 is ascribed to the redox reactions of the 9,10-anthraquinone moiety in free ARS and ARS-PBS. 

**Figure 6:**
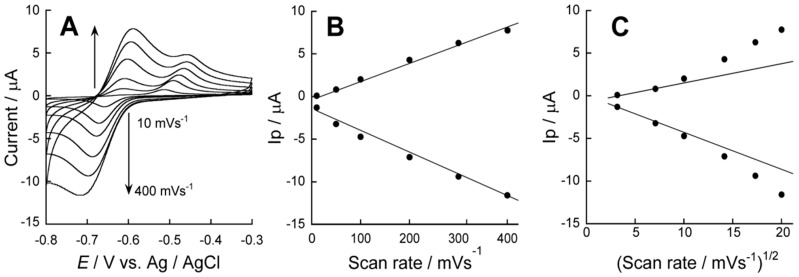
(**A**) The effect of the scan rate on the CV of the ARS-confined (PBA-PEI/CMC)_10_PBA-PEI film-coated electrode at pH 9.0; (**B**) plots of the anodic and cathodic peak current (Ip) at −0.6 to −0.7 V of the CV vs. scan rate; and (**C**) plots of the peak current of the CV vs. square root of scan rate.

Figure 6A shows the CV of the ARS-confined (PBA-PEI/CMC)_10_PBA-PEI film-coated electrode recorded at a scan rate of 10–400 mV∙s^−1^. The anodic and cathodic peak currents at −0.6 to −0.7 V depended almost linearly on the scan rate (Figure 6B) while the plots of the peak currents vs. square root of the scan rate deviated from the straight line (Figure 6C), suggesting that the redox reactions of ARS are a surface process. Similarly, the redox behavior of ARS has been reported in PBA-modified polymer film-coated electrodes [25].

**Figure 7:**
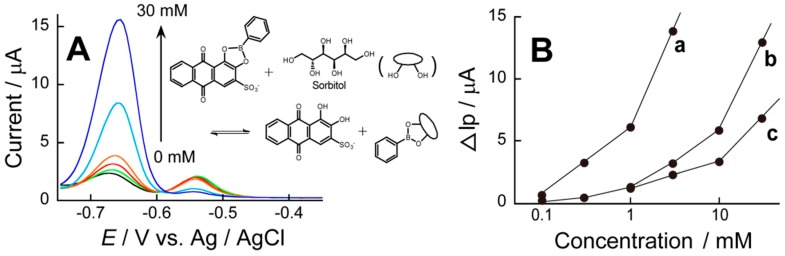
(**A**) DPVs of the ARS-confined (PBA-PEI/CMC)_10_PBA-PEI film-coated electrodes in the presence of sorbitol and the binding equilibrium between ARS-PBA and sorbitol (inset); and (**B**) changes in the intensity of peak current in DPVs at ca. −0.67 V as a function of the concentration of L-dopa (a); sorbitol (b); and glucose (c). ∆Ip denotes the peak current around −0.67 V increased in the presence of L-dopa, sorbitol, and glucose. DPVs were recorded at pH 9.0.

The CV and DPV of the ARS-confined (PBA-PEI/CMC)_10_PBA-PEI film-coated electrode may depend on the concentration of diol compounds in the solution because diols bind competitively to the PBA moieties in the film to liberate ARS from the boronate ester. Thus, the oxidation current originating from the ARS-PBA may decrease when diol compounds are added into the solution. Figure 7A shows the DPVs of an ARS-confined (PBA-PEI/CMC)_10_PBA-PEI film-coated electrode in the solutions of 1–30 mM sorbitol. The DPV exhibited two oxidation peaks originating from the ARS-PBA and free ARS at −0.54 and −0.67 V, respectively, in the absence of sorbitol. The peak current at −0.67 V increased when increasing the concentration of sorbitol added, while the oxidation peak at −0.54 V decreased. The results suggest that a significant portion of ARS in the ARS-PBA was replaced by the added sorbitol, which resulted in a decrease in the concentration of the ARS-PBA species and an increase in free ARS. In other words, sorbitol bound to the PBA residues to form sorbitol-PBA esters in the LbL film, as schematically illustrated in Figure 7A. We have separately confirmed that sorbitol did not show redox response on the ARS-free (PBA-PEI/CMC)_10_PBA-PEI film-coated Au electrode as well as unmodified Au electrode. Thus, sorbitol is not redox active in this potential range. These results support that the redox response reported in Figure 7A originated from the competitive binding of sorbitol to the PBA residues in the LbL film. The addition of L-dopa and glucose also induced similar changes in the DPV of the ARS-confined electrode (Supplementary data).

Figure 7B shows changes in the peak current for free ARS in the DPV as a function of the concentration of sorbitol, L-dopa, and glucose. The oxidation current increased in the presence of 0.3–3 mM L-dopa, while the response to sorbitol and glucose was found in the range 3–30 mM. Thus, the response of the electrode was in the order of L-dopa > sorbitol > glucose, which is in line with the order of the binding constants of PBA to the diol compounds. The binding constants of PBA to L-dopa, sorbitol, and glucose are reported to be 770, 370, and 4.6 M^−1^, respectively [19,26]. These results further confirm that the competitive binding of diol compounds to the PBA moiety is responsible for the redox response of the ARS-confined electrode. Unfortunately, the calibration graphs are not linear vs. the concentrations of target compounds. This should be addressed to improve the performance of the sensors. 

## 4. Conclusions

The results suggest the possible use of ARS-confined PBA-PEI/CMC LbL film-coated electrodes for the construction of voltammetric sensors for diol and polyol compounds. The voltammetric measurements were carried out without adding any reagent in the sample solution by using PBA-PEI as film component. However, the reusability of the electrode must be further improved. The voltammetric response slightly decreased after repeated measurements, owing to the leakage of ARS from the LbL film. This may be solved by constructing LbL films using ARS-modified polymers. In addition, the preparation of the modified electrode is somewhat time-consuming. The processes for the preparation of LbL films and adsorption/rinsing of ARS could be improved by optimizing the experimental conditions.

## Supplementary Materials

The following are available online at http://www.mdpi.com/1424-8220/18/1/317/s1.
