# Supplementary material for: Voltammetric Response of Alizarin Red S-Confined Film-Coated Electrodes to Diol and Polyol Compounds: Use of Phenylboronic Acid-Modified Poly(ethyleneimine) as Film Component
Source: Sensors (Basel). 2018 Jan 22;18(1):317. doi: 10.3390/s18010317 (PMC5795570; doi:10.3390/s18010317)
Supplement: Supplementary file 1 [file sensors-18-00317-s001.pdf]

Supplementary data

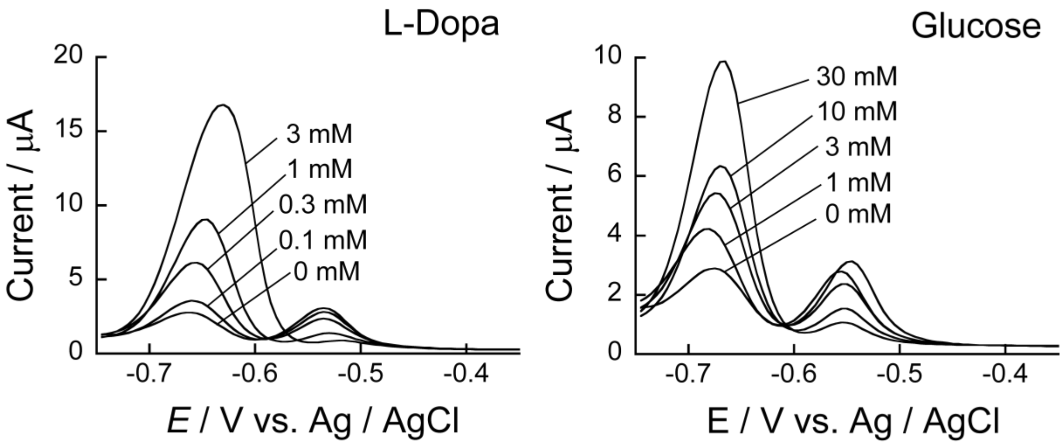

DPVs of the ARS-confined (PBA-PEI/CMC)$_{10}$PBA-PEI film-coated electrodes in the absence and presence of L-dopa (0.1-3 mM) and glucose (1-30 mM) at pH 9.0.
